# Supplementary material for: SARS-CoV-2 transmission in students of public schools of Catalonia (Spain) after a month of reopening
Source: PLoS One. 2021 May 12;16(5):e0251593. doi: 10.1371/journal.pone.0251593 (PMC8115831; doi:10.1371/journal.pone.0251593)
Supplement: Attachment — Submitted filename: response_letter.docx [file pone.0251593.s003.docx]

Dear editor,

We are pleased to submit a revised version of the manuscript PONE-D-21-02405: “SARS-CoV-2 transmission in students of public schools of Catalonia (Spain) after a month of reopening”.

In the revised version of the manuscript, we address the questions raised during the review process, which we describe in the rest of the present letter.

Thank you for your comments. We think these have contributed to improving the previous version.

Best wishes,

Anna Llupià

_________

Page and line numbers correspond to the version originally submitted.

Journal Requirements

- TItlepage: We have updated the format of the author according to the journal requirements.
- Page 9, line 9 We have removed the sentence “data not show” with the corresponding values of RR and CI for the interaction.
- We have updated the information in the “Financial disclosure” to match with the “Funding information version”. The financial statement has been included also in the cover letter of the revision.

**Additional Editor Comments:**

- *The data however has important limitations that should also limit the strength of the conclusions*.

We have rearranged and rewritten parts of the discussion to highlight the limitations arising from the data available. For instance, the sentence “When this transmission is high, our findings imply that related-to-school infections do commonly occur and get probably transmitted to households through children and teenagers. ” (page 12, lines 22-24 submitted version) has been deleted. Also some emphasised adjectives have been deleted, for instance where said “the incidence in the community in the first two weeks plays **an important role** in the school incidence in weeks 4 and 5.” (page 11, line 10, submitted version) now says “the incidence in the community weeks 1-2 plays **a role** in the school incidence in weeks 4-5.”

Conclusions are now like this: “Safety measures seem insufficient to prevent cases in schools which had reported cases in the previous weeks. The safest way to reopen schools, to protect the health of students, staff and their families seems to improve case and contacts management and reduce transmission in the community to minimal levels [[4]](https://paperpile.com/c/bc5WbB/aDeo), which will be easier when vaccines are available.”

- *Furthermore, the discussion misses in my opinion other references in which school transmission of course reflected community transmission (one of the findings of this article), but didn't contribute so importantly to overall transmission. These should be added to the discussion.*

Our study design does not allow us to draw conclusions on the effect of within-school transmission in general community transmission. As the editor rightly points, the extent to which this within-school transmission contributes to overall transmission is a very important question. We have enlarged the paragraph in the discussion which was formerly lines 14-18 of page 10 (submitted version) to include the paper <https://doi.org/10.1073/pnas.2020834118> together with different views of this important question <https://www.bmj.com/content/372/bmj.n521> and <https://doi.org/10.1016/S0140-6736(21)00622-X>. We have also included a reference to a recent study <https://doi.org/10.1093/cid/ciab228> and the large cohort study <https://www.bmj.com/content/372/bmj.n628> recently published.

**Comments to the Author. Reviewer #1**

- *The existence and the impact of within school transmission can not be inferred with the data available and with this study design. When adjusted by community incidence (BHA), the RR is significant but very mild (RR 1.002).*

The RR of 1.002 refers to the risk of community incidence in the BHA, in terms of cases per 100,000. In lines 10-13 of page 11 (original submission) we state that “The incidence in the community in the first two weeks plays an important role in the school incidence in weeks 4 and 5. Adjusting for the rest of the variables, for every 100 cases increase in the BHA incidence (measured as cases per 100,000 inhabitants in the first two weeks), school incidence increased by around 20%.”. Note that incidence was around 100-200 cases per 100,000 in weeks 1 & 2.

We agree that the impact of within school transmission cannot be inferred from the data available and with this study design. To avoid confusion, we have removed the following sentence from the discussion: “When this transmission is high, our findings imply that related-to-school infections do commonly occur and get probably transmitted to households through children and teenagers” (lines 22-24, page 12 submitted version).

*- Also, the increase in incidence at week 4-5 was higher when the schools had zero cases in the first two weeks compared to those with at least one case (Figure 1).*

In our study we do not evaluate the increase in incidence in weeks 4-5 with respect weeks 1 and 2 and we included Figure 1 to illustrate that incidence in weeks 4-5was higher in schools which had reported cases in weeks 1-2 with respect to those which did not report any, and that this holds disaggregating by community incidence in the area. We agree that the writing of one sentence may induce confusion and we have removed it (lines 13-16 in page 11, submitted version).

Even if we do not analyze the increase in incidence in Figure 1, it is reasonable to expect that the relative growth in incidence in schools which did not report cases in weeks 1-2 (plot in red) is higher with respect to schools which do report cases (plot in green). Indeed, schools which report cases in weeks 1-2 applied contention measures (isolation and quarantines) and, if measures were enough, one would expect that incidence is not higher than in schools which do not report cases. A conclusion of our study is that these contention measures are not enough to stop transmission and the incidence in weeks 4-5 is still higher in schools which report cases in weeks 1-2. The new version of the discussion has incorporated these observations.

*- It does not reject the study and its findings, but the impact of within school transmission should be cautiously stated and discussed better*.

Discussion and conclusions have been rewritten in order to be more cautious with the conclusions of the impact of in-school transmission.

**Minor comments.**

- In the discussion we have included the following sentence to account for the possibility that higher classroom size may explain the increased risk in secondary schools: “A possible explanation is the number of students per class, higher in secondary. “ (page 11, lines 20-21, submitted version).
- *Page 2 / Line 9 – wrong capital letter in To; two "s" in sSARS-CoV-2:* Changed
- *Page 10 / Line 18 - "may have contributed".* Changed.
- *Page 10 / Line 21 – no comma after Spain.* Changed.
- *Page 11 / Line 19 – ineffective.* Changes from “infective” to “infectious”.
- *Page 11 / Lines 25-26 – confusing sentence; suggest rewriting.* We have changed the writing and we now think that it is clearer together with an extra sentence in the same paragraph (see version with tracked changes).
- *Page 12 / Line 2 – SARS-COV-2.* Changed
- *Page 13 – paragraph starting in line 5 is too long and hard to read*. It has been reorganized.

**Comments to the Author. Reviewer #2**

- *Please review line 9 page 4:* We have corrected the two misprints.
- *If possible, it would be interesting to consider impact of vaccination in these process.* We have updated the sentence in the last paragraph adding “which will be easier when vaccines are available.” Note that during the study period (ending in mid-October 2020, vaccination had not yet been rolled out).

**Comments to the Author. Reviewer #3**

*Simple analysis and it is not described in sufficient detail. The study presents the results of original research, but the analysis must be describe in sufficient detail.*

We have revised the methods section to clarify some points of the analysis description. We used a multilevel Poisson regression with random effects at the Sanitary Region and BHA levels, with an offset at the number of students minus number of registered students per school excluding the cases from the first two weeks. All the analysis and datasets are available as supplementary files.

*Also, the conclusions are not presented in an appropriate fashion.*

Conclusions are now like this: “Safety measures seem insufficient to prevent cases in schools which had reported cases in the previous weeks. The safest way to reopen schools, to protect the health of students, staff and their families seems to improve case and contacts management and reduce transmission in the community to minimal levels [[4]](https://paperpile.com/c/bc5WbB/aDeo), which will be easier when vaccines are available.”

The conclusions of the abstract have been rewritten accordingly.

*The authors must highlight the limitation of this study related to data were aggregated at the school level and it was not possible to know if different SARS-CoV2 infection cases shared the same place or extracurricular activities or which was their age group.*

We agree that it is important to highlight the limitation of the study related to the aggregation of data at school level and not classroom level and the study design. To emphasize this, we have removed the sentence “When this transmission is high, our findings imply that related-to-school infections do commonly occur and get probably transmitted to households through children and teenagers” and we have rewritten the

paragraph of limitations (page 12, lines 1-18 in the original submission).
